# A Case of BRASH (Bradycardia, Renal Dysfunction, Atrioventricular Node Blockade, Shock, and Hyperkalemia) Syndrome Following Initiation of a Sodium-Glucose Cotransporter-2 (SGLT-2) Inhibitor and a Loop Diuretic

**DOI:** 10.7759/cureus.62830

**Published:** 2024-06-21

**Authors:** Christian Wright, Filip Saitis, Wadah Ayoub, Noah R Schneegurt

**Affiliations:** 1 Department of Internal Medicine, Mount Carmel Health System, Columbus, USA; 2 Department of Medical Education, Medical College of Wisconsin, Milwaukee, USA; 3 Department of Nephrology, Medical College of Wisconsin, Milwaukee, USA

**Keywords:** renal failure, heart failure, shock, loop diuretic, sglt-2 inhibitor, bradycardia, brash syndrome

## Abstract

BRASH (bradycardia, renal dysfunction, atrioventricular node blockade, shock, and hyperkalemia) syndrome is a recently recognized clinical process that can be fatal if not adequately and promptly treated. As such, it is important for clinicians to recognize the syndrome. This case demonstrates an example of BRASH syndrome in a 73-year-old patient with heart failure occurring after initiation of dapagliflozin, a drug not previously associated with this phenomenon in the literature. Given the increasingly appreciated clinical utility of sodium-glucose cotransporter-2 (SGLT-2) inhibitors, prescribers must respect their potential side effects in patients with underlying comorbidities and remember the importance of re-evaluating renal function after initiation of these medications. Here, we review the pathophysiology of BRASH, the renal effects of SGLT-2 inhibitors, and the importance of educating patients on volume management and diuretic dose titration at home.

## Introduction

This article was previously presented as a meeting abstract and poster at the 2024 Society of Critical Care Medicine (SCCM) Ohio Virtual Research Day on June 6, 2024.

BRASH (bradycardia, renal dysfunction, atrioventricular node blockade, shock, and hyperkalemia) syndrome is gaining recognition as a distinct clinical phenomenon with potentially fatal consequences, including cardiac arrest and death, if not promptly treated [1]. Possible precipitants are numerous and include any entity that can lead to kidney injury or hyperkalemia. It is important that clinicians be able to identify cases of BRASH; however, it is also important that they recognize possible triggers of this syndrome, patients at risk of developing it, and methods to avoid the hemodynamic mechanisms that underpin its pathophysiology. We present a case of BRASH following the initiation of a sodium-glucose cotransporter-2 (SGLT-2) inhibitor and maintenance loop diuretics. The purposes of this report are to demonstrate an example of BRASH syndrome, explore the renal effects of SGLT-2 inhibitors, and review patient education on diuresis and volume management in the outpatient setting.

## Case presentation

A 73-year-old male presented to the emergency department via EMS with reports of lightheadedness, malaise, and anuria for approximately 48 hours. The patient also reported that his left hand felt cold and weak. He denied any numbness or paresthesias in his hand.

Past medical history included hypertension, heart failure with preserved ejection fraction, type 2 diabetes mellitus with diabetic nephropathy, and stage III chronic kidney disease. He was on carvedilol for the management of his heart failure and hypertension. Of note, the patient had recently been admitted to the hospital with decompensated heart failure and was discharged 10 days prior to the above presentation. He was treated with diuretics and monitored for stability of renal function. Upon discharge, he was prescribed an equivalent diuretic dose of torsemide 20 mg twice daily to begin maintenance diuretic therapy and dapagliflozin 5 mg daily. Additional pertinent medications included amlodipine 10 mg daily, chlorthalidone 25 mg daily, metformin 1,000 mg twice daily, aspirin 81 mg daily, and atorvastatin 40 mg daily.

Upon EMS arrival at the scene, the patient was noted to have a heart rate in the range of 30-40 beats per minute. Atropine was administered with partial improvement of his symptoms. On presentation to the emergency department, initial vital signs revealed a heart rate of 45 beats per minute with a blood pressure of 110/63. The respiratory rate was 19 breaths per minute, and the patient had an oxygen saturation of 98% on ambient air. However, vital signs demonstrated marked variability with intermittent symptomatic bradycardia and hemodynamic instability. The patient's heart rate and blood pressure ceased to improve with atropine administration in the emergency department.

**Figure 1:**
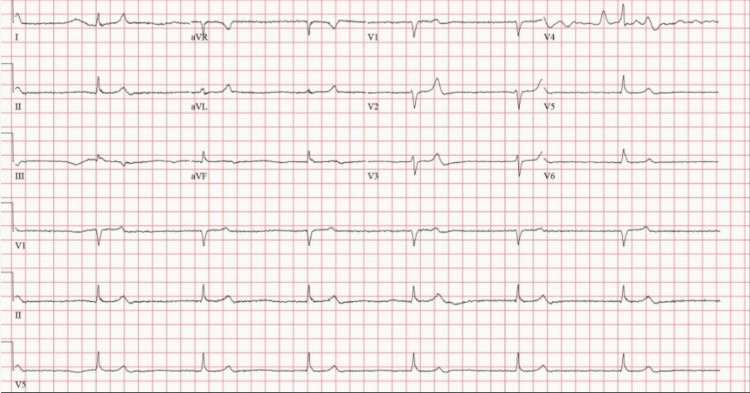
This patient’s electrocardiogram demonstrated junctional bradycardia due to sinoatrial node dysfunction with a heart rate of 40.

The initial electrocardiogram demonstrated junctional bradycardia with a heart rate of 40, as evidenced by Figure 1. 

Laboratory studies revealed a potassium of 7.4 mmol/L, a creatinine of 9.32 mg/dL, and a blood urea nitrogen concentration of 88 mg/dL. The bicarbonate concentration was 20 mmol/L, and the pH of venous blood gas was 7.33. Of note, baseline creatinine was approximately 2.20 mg/dL. Intravenous insulin, sodium bicarbonate, and calcium gluconate were administered, as well as inhaled albuterol and oral potassium binding agents. The patient was subsequently admitted to the intensive care unit. Nephrology was consulted for assistance. Carvedilol, dapagliflozin, and torsemide were held, and intravenous fluids were initiated. A temporary hemodialysis catheter was placed, and hemodialysis was initiated.

The patient’s vital signs and electrolyte derangements subsequently improved. His reports of left-hand weakness and coldness resolved after the above therapy. His renal function gradually recovered over the course of several days, and hemodialysis was discontinued. The patient was ultimately discharged from the hospital with a resumption of his torsemide at a reduced dose of 20 mg once daily. However, his SGLT-2 inhibitor was not resumed at the time of discharge. 

## Discussion

The BRASH syndrome is gaining recognition as a distinct clinical phenomenon. The findings in BRASH syndrome are interrelated via a pathophysiologic cycle in which a renal insult leads to the development of hyperkalemia. Hyperkalemia, in combination with AV node blocking agents, some of which rely on renal clearance, increases the propensity for bradycardia, which can become hemodynamically significant and thus worsen the renal injury, further fueling the cycle [1].

Any factor that can cause acute kidney injury or hyperkalemia can theoretically lead to BRASH syndrome in a patient who is otherwise at risk. In the case described here, there was likely a multifactorial explanation for the patient’s presentation. Initiation of a loop diuretic and excessive diuresis possibly contributed through hypovolemia and pre-renal kidney injury. This is a well-documented complication of diuretics [2]. Similarly, many other medications are widely known to directly reduce renal function, including those in the class of renin-angiotensin system blockers. Another such class of medications is the SGLT-2 inhibitors [3], though this might not be as readily appreciated by prescribers.

With the increasing utilization of SGLT-2 inhibitors, providers must note potential side effects and the general physiologic impact of these agents. While studies show that they provide overall renal and cardiovascular benefits, research has also provided evidence of an initial drop in creatinine clearance of 2-5 mL/min, similar in magnitude to that seen with angiotensin-converting enzyme (ACE) inhibitors [3,4]. Though this is often well-tolerated in a patient with intact renal function, those with chronic kidney disease and those who are on other renal-altering medications might require closer monitoring, as such a drop is a larger portion of the existing kidney function and can predispose these patients to complications. In the case presented here, it was felt that the patient’s baseline renal disease, a new diuretic, and a new SGLT-2 inhibitor all had cumulative effects and incited his acute kidney injury.

Notably, the patient was diuresed adequately in the hospital and monitored until his creatinine was stabilized. Subsequently, he was transitioned to the equivalent dose of oral torsemide for ongoing volume management. Considering his stable renal function on the newly prescribed diuretic, it is important to remember that the reduction in creatinine clearance associated with SGLT-2 inhibitors tends to occur over several weeks [3]. For some individuals, this mild renal impact can gradually lead to intolerance of other medications, possibly necessitating dose modifications where clinically appropriate.

Additionally, this report emphasizes the importance of patient education regarding diuretic dose titration at home to avoid side effects on one end of the spectrum and hypervolemia on the other end. This is particularly prescient in the setting of increasing pressures to discharge patients in a timely manner from hospital care, which carries the risk of hindering proper discharge optimization [5]. The potential side effects that diuretics pose for a patient population already at risk from multiple comorbidities can be at least partly mitigated by appropriate and thorough education.

One study observed patients diagnosed with heart failure who were engaged in a home-monitoring program. It directly compared the patients who were eventually hospitalized with those who were not. The study found that the patients who required hospitalization experienced weight gain in the 30 days leading up to their admission, most profoundly in the week preceding admission [6]. Patients who are prescribed diuretics may benefit from detailed education regarding at-home diuretic dose titration based on fluctuations in daily body weight. Another study tracked patients prescribed furosemide for a diagnosis of heart failure over a 17-month period. It found that patients who were given additional education on the use of diuretics using visual aids experienced fewer hospitalizations and incidences of death than a control group who were given a standard informational booklet on the treatment of heart failure. The training involved a one-hour session instructing patients how to adjust their diuretic dose according to daily weight trends, with increased doses for daily weight gain and decreased doses for daily weight loss. Each patient was given a scale and a booklet and received telephone follow-up calls to aid with adherence. The study found that of the 127 randomized patients, 61% of the control group experienced at least one hospitalization or died, compared to only 42% in the intervention group [7]. Such studies support a more detailed approach to instructing patients on at-home volume status management to avoid re-admissions for heart failure exacerbations and to prevent hypovolemic complications of loop diuretics.

## Conclusions

In conclusion, BRASH syndrome is still in the early stages of clinical recognition, but it is a possible result of ordinary heart failure therapy, particularly in patients with pre-existing renal disease. This case uniquely highlights the potential for even modest reductions in renal function caused by SGLT-2 inhibitors to contribute to BRASH syndrome in patients with chronic kidney disease, particularly when combined with diuretics. Clinicians should carefully consider the potential risks and benefits of SGLT-2 inhibitors in this patient population and make any necessary adjustments to their monitoring. Additionally, education on diuretic dose titration and volume management has been shown to be beneficial in patients with heart failure to avoid both over-diuresis and admission for heart failure exacerbation.
